# Supplementary material for: Deep learning methods to forecasting human embryo development in time-lapse videos
Source: PLoS One. 2025 Sep 2;20(9):e0330924. doi: 10.1371/journal.pone.0330924 (PMC12404471; doi:10.1371/journal.pone.0330924)
Supplement: S1 Fig — ConvLSTM processes an input matrix (Xt) by modeling spatial distribution with temporal dependencies. ConvLSTM achieves this task through element-wise multiplication (hadamard product) of the feature vector of Xt with the LSTM states at time t. Xt corresponds to a video frame at t. (PDF) [file pone.0330924.s001.pdf]

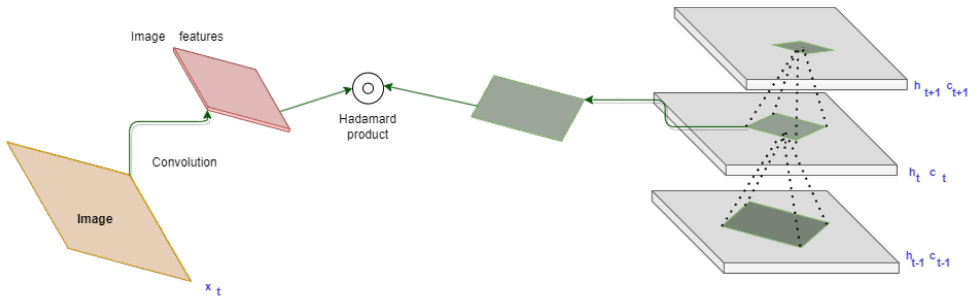


**S1 Fig. Inner structure of Convolutional LSTM (ConvLSTM)** For an input matrix($X_t$), ConvLSTM models the spatial domain information with temporal dependencies on the element wise multiplication (hadamard product) between image's feature vector and LSTM states at time t. $X_t$ is a frame representing video sequence at time t.
