# Supplementary material for: Deep learning methods to forecasting human embryo development in time-lapse videos
Source: PLoS One. 2025 Sep 2;20(9):e0330924. doi: 10.1371/journal.pone.0330924 (PMC12404471; doi:10.1371/journal.pone.0330924)
Supplement: S2 Fig — The videos used for training and evaluating the AI system contained distributions of embryo development stages as: Part a): Cell stages in the transfer videos for the cells stage study. Part b): Cell stages in the avoid videos for the cells stage study. Part c): Embryo development stages in the transfer videos for the blastocyst study. Part d): Embryo development stages in the avoid videos for the blastocyst study. (PDF) [file pone.0330924.s002.pdf]

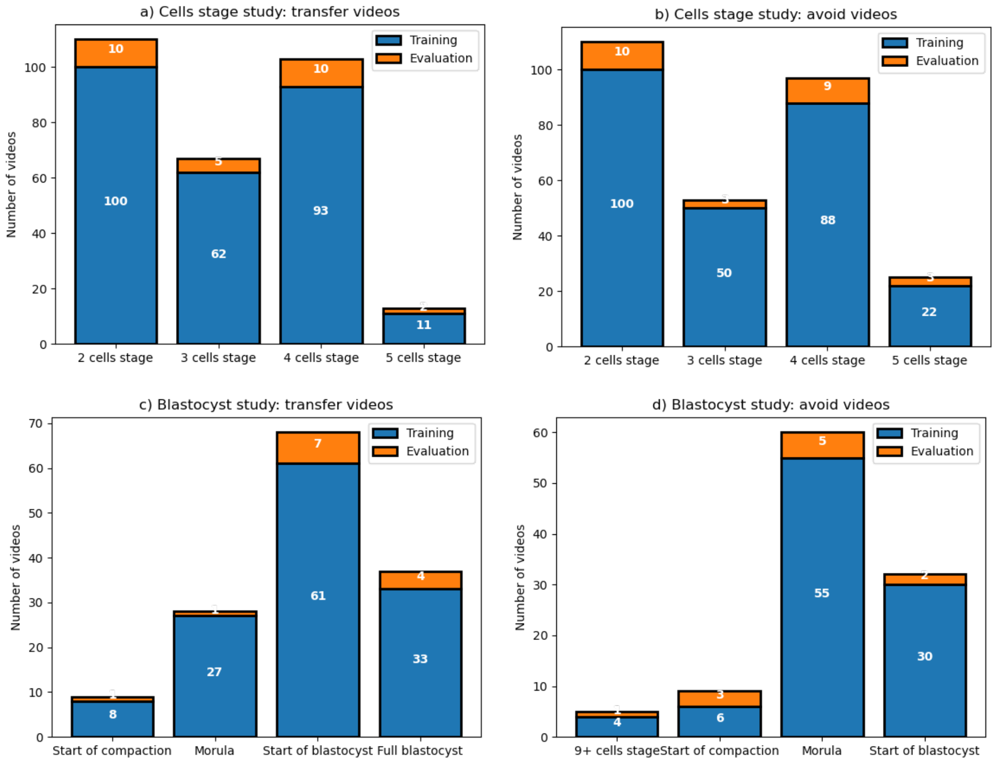

S1 Fig. Embryo cell stages distribution in the datasets. The videos used for training and evaluating the AI system contained distributions of embryo development stages as: Part a): Cell stages in the transfer videos for the cells stage study. Part b): Cell stages in the avoid videos for the cells stage study. Part c): Embryo development stages in the transfer videos for the blastocyst study. Part d): Embryo development stages in the avoid videos for the blastocyst study.
