# Supplementary material for: Deep learning methods to forecasting human embryo development in time-lapse videos
Source: PLoS One. 2025 Sep 2;20(9):e0330924. doi: 10.1371/journal.pone.0330924 (PMC12404471; doi:10.1371/journal.pone.0330924)
Supplement: S6 Fig — a) A transfer video from the independent set (evalBT). b) and c) Avoid videos from the independent set (evalBA). The actual (ground truth) frames are on the left, while the predicted frames are on the right, with time points highlighted in blue and reported in hours post insemination. (PDF) [file pone.0330924.s006.pdf]

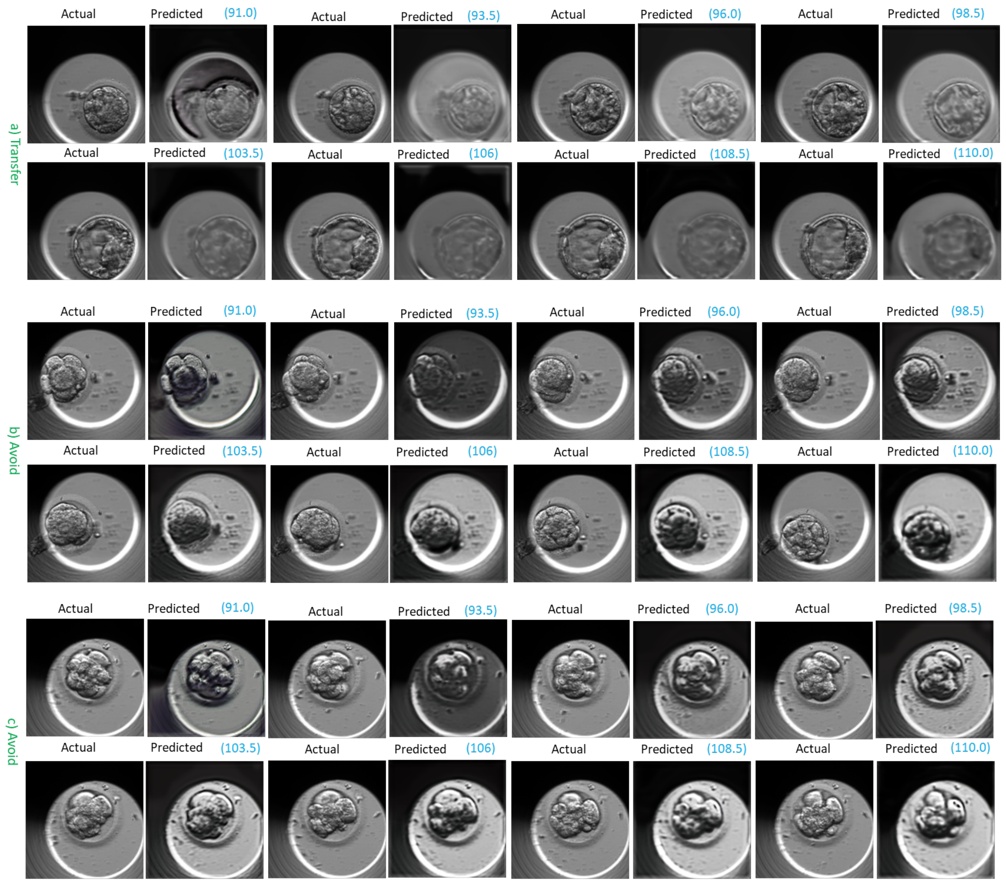

S6 Fig. Blastocyst study: forecasted embryo development at multiple time points with the 'Forecasting the Next 7 Frames' strategy. a) A transfer video from the independent set (evalBT). b) and c) Avoid videos from the independent set (evalBA). The actual (ground truth) frames are on the left, while the predicted frames are on the right, with time points highlighted in blue and reported in hours post insemination.
