# Supplementary material for: Deep learning methods to forecasting human embryo development in time-lapse videos
Source: PLoS One. 2025 Sep 2;20(9):e0330924. doi: 10.1371/journal.pone.0330924 (PMC12404471; doi:10.1371/journal.pone.0330924)
Supplement: S1 Appendix — (PDF) [file pone.0330924.s007.pdf]

S1 Appendix. Recurrent neural network: information processing, mathematical definitions and formulas

Recurrent neural network

A recurrent neural network (RNN), is capable of processing sequence of data. For a video sequence X_i at current time step t, RNN predicts the next frame at step t+1. For the prediction task, RNN utilizes the information from current frame at t and from the previous frames at earlier time steps (0...t-3, t-2, t-1). The RNN achieves this through the concept of hidden state, which allows the network to retain and recall prior information at t. A RNN consists of multiple fixed activation function units corresponding to a specific time step, with each unit having a hidden state. Each hidden state share the same weight W connecting the units across the network. The processing of a video sequence and the working principle of RNN is shown in Fig 1. The

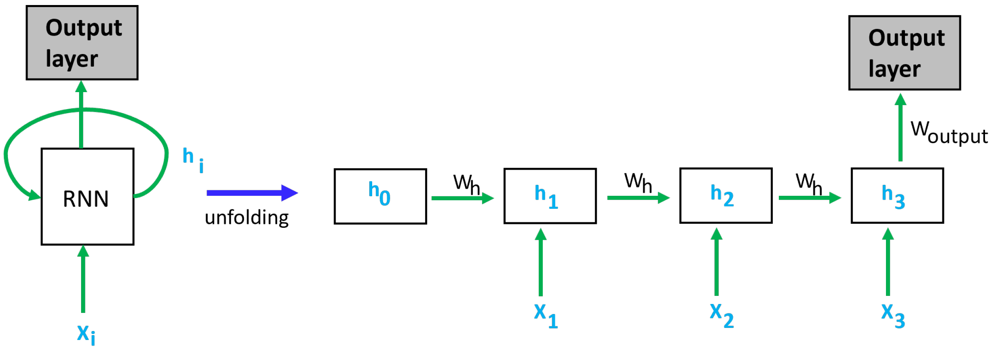

Recurrent Neural Network. The RNN processing the video sequence X_i is unfolded as sequential update to the RNN's hidden vector h_i with each video frame (X_1, X_2, X_3) processing and using the same connecting weight W_h shared across the RNN. After processing the sequence, RNN passes the information to the output layer.

information within a unit is updated when the hidden vector loops back to incorporate the current input value with previously stored values using the equation Eq (1). Here, h(t) is the hidden vector at current time step input $x_{(t)}$. the term f represents activation function with the weights W_{xh} as the weights from input to the hidden unit and W_{hh} as the weights from hidden to hidden units. The term b_h represents bias of the hidden unit.

$$h(t) = f(W_{xh}^T x(t) + W_{hh}^T h(t-1) + b_h) \qquad (1)$$

The formula for calculating output $\hat{y(t)}$ is defined by the equation Eq (2). In the equation, W_o represents the weight at output layer and b_o is the bias at output layer.

$$\hat{y(t)} = f(W_o^T h(t) + b_o) \qquad (2)$$
