# Supplementary material for: Deep learning methods to forecasting human embryo development in time-lapse videos
Source: PLoS One. 2025 Sep 2;20(9):e0330924. doi: 10.1371/journal.pone.0330924 (PMC12404471; doi:10.1371/journal.pone.0330924)
Supplement: S2 Appendix — (PDF) [file pone.0330924.s008.pdf]

S2 Appendix. Long short-term memory: Information processing, mathematical definitions and formulas

Long short-term memory

The Long short-term memory (LSTM) is a type of recurrent neural network with additional memory cells for processing longer sequences. A LSTM unit has two states: hidden state (h_t) and cell state (c_t). At a current time step t, LSTM uses h_t to retrieve information from previous time step (0...t-3, t-2, t-1) and c_t for calculating the output. The LSTM processes single time step using three gates: input gate, forget gate and output gate. The gates should be considered as binary classifier or logistic regression neurons carrying forward information from the previous time step $(t-1)$. The forget gate fg_t controls if the information from $t-1$ should be used or be forgotten. The input gate ig_t adds new or updates the current information at t. The output gate og_t passes the added or updated information from t to $t+1$. Additionally, fg_t and ig_t manages c_t, and og_t is responsible for updating h_t. At any time step, to retrieve LSTM's output, we can apply softmax activation on the corresponding hidden state. A block diagram explaining the architecture and workflow of LSTM processing a video sequence is shown in Fig 1.

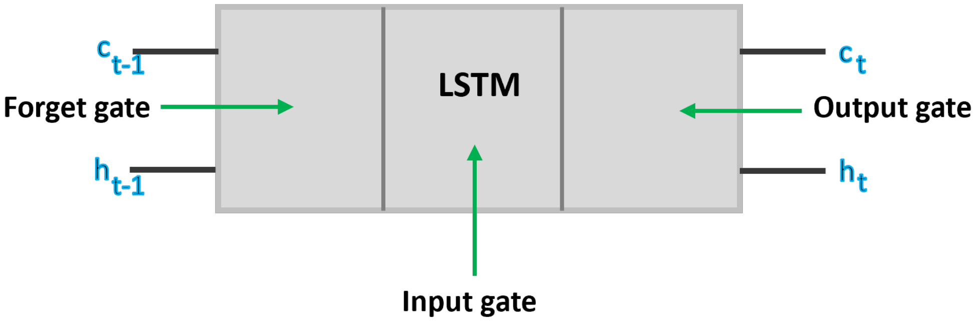

Long short-term memory (LSTM) For a video sequence at current time step t, the first part in LSTM with forget gate decides whether or not to include the sequence information at previous time step $t-1$. In the second part, the input gate of LSTM controls the learning of the sequence information at the time step t and in the final part the output gate is responsible for passing the processed information to next time step $t+1$. With each time step, LSTM's cell state (c_t) and the hidden state (h_t) are updated.

LSTM: forget gate

The forget gate (fg_t) controls the information flow from $t-1$ using the equation Eq (1). Here, W_{xfg} represents the weight between the input and fg_t. W_{hfg} is the weight between h_t and fg_t. The term h_{t-1} corresponds to hidden unit from previous time step and b_{fg} is the bias. The term x_t represents input at t. The sigmoid function σ ensures that the output is between 0 and 1.

$$fg_t = \sigma(W_{xfg}^T x_t + W_{hfg}^T h_{t-1} + b_{fg}) \quad (1)$$

LSTM: input gate

The input gate (ig_t) adds information using the equation Eq (2). Here, x_i represents input at t and W_{xig} represents the weights between this input and ig_t. The term W_{hig} represents the weight between the h_t and the ig_t. The term b_{ig} represents the bias and sigmoid function σ brings the output between 0 and 1.

$$ig_t = \sigma(W_{xig}^T x_i + W_{hig}^T h_{t-1} + b_{ig}) \tag{2}$$

LSTM: output gate

The output gate og_t passes information between two LSTM's unit using the equation Eq (3). Here, W_{xog} represents the weights between current input x_i and og_t and W_{hog} represents the weight between h_t and og_t. The term b_{og} represents the bias and the sigmoid function σ pushes the output between 0 and 1.

$$og_t = \sigma(W_{xog}^T x_i + W_{hog}^T h_{t-1} + b_{og}) \tag{3}$$

LSTM: cell state

The cell state (c_t) is updated by fg_t and the ig_t through the equation Eq (4). Here, W_{xc} represents the weights between the input x_t and c_t, W_{hc} represents the weight between the h_t and c_t, The term b_c is the bias and the operator $\odot$ represents element-wise multiplication or hadamard product.

$$c_t = fg_t \odot c_{t-1} + ig_t \odot \tanh(W_{xc}^T x_t + W_{hc}^T h_{t-1} + b_c) \tag{4}$$

Using the equation Eq (1), we know fg_t is between 0 and 1. Thus, if $fg_t = 0$, the hadamard product = 0 and c_t forgets the previous cell state. But if $fg_t = 1$, $c_{t-1} = 1$ and c_t retains the previous cell state. For ig_t, the tanh activation brings the value between -1 and 1, so depending upon the sign, the update to c_t occurs.

LSTM: hidden state

The hidden state of LSTM is updated by the output gate og_t using the equation Eq (5). Due to hadamard product, og_t decides how c_t passes onto h_t.

$$h_t = og_t \odot \tanh(c_t) \tag{5}$$
