# Supplementary material for: Deep learning methods to forecasting human embryo development in time-lapse videos
Source: PLoS One. 2025 Sep 2;20(9):e0330924. doi: 10.1371/journal.pone.0330924 (PMC12404471; doi:10.1371/journal.pone.0330924)
Supplement: S3 Appendix — (PDF) [file pone.0330924.s009.pdf]

S3 Appendix. Convolutional long short-term memory: Information processing, mathematical definitions and formulas

Convolutional long short-term memory

Convolutional long short-term memory (ConvLSTM) unit has hidden state and cell state. The unit uses forget gate, input gate and output gate to process information across the unit. The states and gates in ConvLSTM are represented as 3D tensors. In below equations $*$ denotes convolution operation and $\odot$ denotes element-wise multiplication or hadamard product.

ConvLSTM: forget gate

The forget gate (fg_t) uses the equation Eq (1) to incorporate previous time step $t-1$ information at the current time step t. Here, W_{xfg} represents the weight between the input x_t and fg_t. The term W_{hfg} is the weight between h_t and fg_t. h_{t-1} is the previous time step's hidden unit and W_{cfg} is the weight between c_t and fg_t. c_{t-1} is the previous time step's cell state and b_{fg} is the bias. The sigmoid function σ ensures that the output is between 0 and 1.

$$fg_t = \sigma(W_{xfg}^T * x_t + W_{hfg}^T * h_{t-1} + W_{cfg}^T \odot c_{t-1} + b_{fg}) \tag{1}$$

ConvLSTM: input gate

The input gate (ig_t) adds information at the t using the equation Eq (2). Here, W_{xig} represents the weights between the input x_i and ig_t. The term W_{hig} represents the weight between the h_t and ig_t, h_{t-1} is the previous time step's hidden unit. W_{cig} is the weight between c_t and ig_t, c_{t-1} is the previous time step's cell state and b_{ig} is the bias. The sigmoid function σ brings the output between 0 and 1.

$$ig_t = \sigma(W_{xig}^T * x_i + W_{hig}^T * h_{t-1} + W_{cig}^T \odot c_{t-1} + b_{ig}) \tag{2}$$

ConvLSTM: output gate

The output gate og_t passes information between two LSTM's unit using the equation Eq (3). Here, W_{xog} represents the weights between current input x_i and og_t. The term W_{hog} represents the weight between h_t and og_t, h_{t-1} is the hidden unit from previous time step. The term W_{cog} is the weight between c_t and og_t, c_t is the current cell state and b_{og} is the bias. The sigmoid function σ pushes the output to be a number between 0 and 1.

$$og_t = \sigma(W_{xog}^T x_i + W_{hog}^T h_{t-1} + W_{cog}^T \odot c_t + b_{og}) \tag{3}$$

ConvLSTM: cell state

The cell state (c_t) is updated by fg_t and the ig_t through the equation Eq (4). Here, W_{xc} represents the weights between the input x_t and c_t, W_{hc} represents the weight between the h_t and c_t, The term h_{t-1} is the hidden unit from previous time step and b_c is the bias.

$$c_t = fg_t \odot c_{t-1} + ig_t \odot \tanh(W_{xc}^T * x_t + W_{hc}^T * h_{t-1} + b_c \quad (4)$$

Using the equation Eq (1), we know fg_t is between 0 and 1. Thus, if $fg_t = 0$, then $c_{t-1} = 0$ and c_t forgets the previous cell state. But if $fg_t = 1$, then $c_{t-1} = 1$ and c_t copies the previous cell state. For ig_t, the tanh activation brings the value between -1 and 1, so depending upon the sign c_t is updated.

ConvLSTM: hidden state

The hidden state of LSTM is updated by the output gate og_t using the equation Eq (5). Due to hadamard product, og_t decides how c_t passes onto h_t.

$$h_t = og_t \odot \tanh(c_t) \quad (5)$$
